# Exploring pathways to Hospital Care for Patients with Alzheimer’s disease and related dementias in rural South Western Uganda

**DOI:** 10.1186/s12913-020-05365-5

**Published:** 2020-06-03

**Authors:** Nathan Kakongi, Godfrey Zari Rukundo, Bizu Gelaye, Edith K. Wakida, Celestino Obua, Elialilia S. Okello

**Affiliations:** 1 Department of Biochemistry, Faculty of Medicine, Mbarara University of Science and Technology, Mbarara, Uganda; 2 Department of Psychiatry, Mbarara University of Science and Technology, Mbarara, Uganda; 3 Department of Epidemiology, Harvard T. H. Chan School of Public Health, Boston, MA USA; 4 The Chester M. Pierce, MD Division of Global Psychiatry, Massachusetts General Hospital, Boston, MA USA; 5 Office of Research Administration, Mbarara University of Science and Technology, Mbarara, Uganda; 6 Department of Pharmacology and Therapeutics and Vice Chancellor, Mbarara University of Science and Technology, Mbarara, Uganda; 7 Mwanza Intervention Trials Unit, Mwanza, Tanzania

**Keywords:** Alzheimer’s disease and related dementias, Caregiver, Dementia, Pathways to health care and southwestern Uganda

## Abstract

**Background:**

In order to analyze use of health services and identify sources of delays in accessing the right care for patients with Alzheimer’s disease and related dementias (AD/ADRD), understanding of care seeking pathways is needed. The objectives of this study were: (i) to explore pathways to hospital care for patients with AD/ADRD and (ii) to describe challenges experienced by the patients and their families while seeking health care.

**Methods:**

Using purposive sampling, 30-in-depth, semi-structured interviews were conducted among caregivers of older adults diagnosed with dementia from rural Southwestern, Uganda. Data was analyzed using ATLAS. Ti software.

**Results:**

There was variability in pathways to care from individual to individual. There was one broader theme captured: points of care choice with four broader categories: hospitals, clinics, places of religious worship and traditional healers’ shrines, each with its facilitating factors, outcomes and challenges encountered. Most of the respondents reported use of hospitals at first and second visit to the health care point but places of religious worship became more common from third to sixth health care encounter. Major improvements (58.1%) were observed on hospital use but little or no help with prayers, clinics and traditional healers. The challenges experienced with formal points of care focused on lack and cost of prescribed drugs, weakening effect of the drugs, lack of skills to manage the condition, and lack of improvement in quality of life. These challenges together with knowledge gap about the disease and belief in spiritual healing facilitated the shift from formal to informal health care pathways, more particularly the places of religious worship.

**Conclusions:**

Our study findings indicate that caregivers/families of patients with dementia went to different places both formal and informal care settings while seeking health care. However, hospital point of care was more frequent at initial health care visits while places of worship took the lead at subsequent visits. Although no specific pathway reported, most of them begin with hospital (formal) and end with non-formal. We recommend that health systems carry out public awareness on dementia.

## Background

Alzheimer’s disease (AD) is the most common form of dementia in older adults accounting for 60–80% of all cases of dementia [1]. It has been reported that AD affects 2% of the population in industrialized countries [2], more than 10% of the population over the age of 65, and 50% of the population over the age of 85 [3]. Currently dementia is estimated at 47 million people worldwide, with nearly 9.9 million new cases reported each year [4] with 60% of the new cases coming from low- and middle-income countries (LMICs) [5]. The disease burden doubles every two decades worldwide but doubles in every 7.2 years in sub-Saharan Africa [6]. In Uganda, data concerning the burden of AD and other dementias is scanty but one study reported that 5.5% of all older adults aged 60 years and above admitted to non-psychiatric wards had dementia [7] .

Health services in Uganda are delivered by public, private for profit (PFP) and private not-for-profit (PNFP) facilities at the level of clinics, health centers and hospitals. The Uganda’s Ministry of Health strategic plan [8] stipulates that specialized clinical services for mental health disorders are only provided at regional referral hospitals. These regional referral hospitals receive patients from the lower health facilities such as clinics, health centers and district hospitals. The clinics (lower levels) are privately owned and managed; largely dispensing medications to individuals for a fee, and also can provide basic outpatient services. District hospitals, also known as general hospitals support all referrals from health centers and clinics; and offer a range of preventive and curative outpatient services, inpatient care, emergency surgery, obstetrics and gynecology, laboratory services, and other general services.

Whereas the public health facilities are expected to provide health services to all people at no cost, the quality of health services delivered in both public and private facilities has been affected by several factors including the distance to health facilities, availability of drugs, equipment, and trained health workers [9]. However, the government through the Ministry of Health [8] has made some attempts to improve the quality of services through building more health facilities, providing more drugs, recruiting more health workers and training health workers through continuing medical education [10]. The health facilities at lower levels (clinics, health centers) and district hospitals do send patients of “complicated diseases” such as dementia to regional referral hospitals which have specialized clinics. However, accessibility to these specialized clinics is another problem itself to rural populations. Also patient satisfaction, cultural and religious attributes of illness are some of the factors that influence whether a person seeks medical advice from formal care settings, complies with treatments and maintains a relationship with the provider/health facility [11] or seeks health care from the informal care settings [12]. Of late some people seek care from religious healers (referred to as places of religious worship in this study) while a small section of the population also seek care from traditional healers (spiritual healers, bone setters, and herbalists) [10].

The mode of care for older adults in Uganda, whether sick or not, is in the hands of family members with a few exceptions of homes for the older adults [13]. Equally, people with dementia are cared for by family members as a collective responsibility but one member is usually the primary caregiver. Like in other countries such as Ghana, South Africa and Tanzania women tend to be pro-active caregivers for people with dementia [14–16], with men providing finances and the role of decision-making about the care [14]. Studies from Ghana and Tanzania have also shown that children and grand-children also take up different components of the responsibility [14, 16].

Available evidence indicates that people with Alzheimer’s disease and or related dementias (AD/RD) seek health care in different ways and meet different challenges along the way [17]. The pathways and challenges vary from individual to individual. According to Carrillo et al., [18] in the healthcare access barriers mode, they categorize factors along the pathways to care to include financial, structural and cognitive. The financial barriers like lack of insurance or money to meet treatment bills limit access to medical treatment [18]; structural barriers like low availability of health facilities and mental health specialists or multi-step care processes tests and meeting specialists reduce accessibility to healthcare; while cognitive barriers like limited knowledge or awareness about the disease influences health seeking behaviors or attitudes [19]. To sum it up, these three types of health care access barriers are associated with decreased screening, late presentation to healthcare, and lack of treatment, which in turn result in poor health outcomes and health disparities among the patients with dementia [18].

Studying the pathways to care is critical to describing health services utilization, characterizing of the sources of delay in attending the right care, and identifying the possible remedies. Prior research shows that these care pathways are not usually random but they are structured by a combination of psychosocial and cultural factors [20]. A study by Nakasujja, Musisi [7] at Mulago national referral hospital in Uganda covered patients who had visited the hospital but did not take into account where these patients passed to reach the hospital and what could affect their path to the facility. Yet, varied beliefs on dementia among family members may cause them seek care in different ways [21]. Most times, care will be sought in different places including faith and traditional healers [22]. A Ugandan study by Nakasujja, Musisi [7] among older adults attending non-psychiatric wards at Mulago National Referral Hospital showed that dementia was second to depression as the most common cause of seeking psychiatric care amongst older adults.

A study in Tanzania found that 41 and 19% of people with dementia had visited Christian faith healers (FHs) and traditional healers (THs) respectively [16]. Another study by Stangeland and colleagues (2008) reported that traditional healers were more available to the communities than medical doctors with a ratio of 1 TH per 350 people and 1 doctor per 33,000 people [23]. Such a growing trend in the use of religious places of worship for spiritual healing over the last 30 years has made it a relatively new type of alternative care, coming to prominence across sub-Saharan Africa [21]. Also the study by Mushi, Rongai [16] in rural Tanzania showed that participants believed their mental problems (including dementia) were caused by either ageing, other chronic diseases, life stresses or witchcraft. This indicates how people’s beliefs in spiritual healing or limited knowledge of dementia influence their health seeking behavior. While such studies have been lacking in Ugandan context, our study explored pathways which these patients of dementia take to reach hospital care, the facilitators for the specific pathways and the challenges experienced by the patients and their families in the process. Our findings will guide the health systems in Uganda and other countries in the regions with similar healthcare systems to accelerate the attention to these people and their family in utilization of formal health services for better health outcomes. The study was carried out in rural southwestern Uganda where most of the older adults live.

## Methods

### Aim and design of the study

This study aimed to explore the pathways undertaken for patients with Alzheimer’s disease and related dementias to reach hospital care and the challenges they meet in the process. It was a descriptive cross-sectional study conducted among caregivers of patients with Alzheimer’s disease and related dementias using in-depth interviews in rural southwestern Uganda.

### Study setting

The study was carried out at homes of recruited participants and psychiatric wards of three referral hospitals of: Mbarara Regional Referral Hospital (MRRH), Kampala International University Teaching Hospital (KIU-TH) and Kabale Regional Referral Hospital (KRRH). The three health facilities are geographically distributed to represent the southwestern region of the country and receive patients from lower health facilities in the region.

### Participants’ recruitment and sampling

The participants were caregivers who had been taking care of older adults (60 years and above) who had been diagnosed with dementia in the formal health care/hospital regardless of their current health condition, previous points of care visited and number of visitations to health care points. The study recruited caregivers who had stayed with the patients for at-least 6 months and above, aged 18 years and above, and agreed to participate in the study by informed consent. Caregivers who were unable to give adequate information due to various reasons like serious sickness or drunkenness were excluded from the study.

Purposive sampling was used to recruit participants from medical records in the psychiatry departments of the three hospitals by medical personnel to get contacts of caregivers of patients who had attended in the last one year and those attending at the time. Those attending at the time were interviewed from the facility while those from past records were contacted by telephone calls and followed up to their homes where they were interviewed from. A total of 30 respondents participated in this study.

### Procedure

In-depth interviews were conducted by the lead author (NK) and two trained research assistants between December, 2018 and January, 2019. Each interview lasted approximately 40 min and was audio recorded. The interviews were conducted in either Runyankore-Rukiga (native language of the area) or English depending on convenience of the respondent, and backed by field notes.

### Data collection and study tools

A semi-structured interview guide was developed by NK in consultation with GZR, BG and ESO. Questions were based on the objectives of the study. The interview guide (Additional file 1) was translated to Runyankore-Rukiga by a native person of the area and back translated to English by one research assistant to ensure consistence. Audio recorders were used to enhance data capture and retrieval to correlate the hand written information. Participants were interviewed to obtain information concerning their pathways to care and challenges met while seeking care. All responses were prompted by open ended questions.

### Data analysis and quality control

Data was transcribed verbatim and translated into English by the two research assistants. The transcripts were read several times by the lead author (NK) and ESO to get familiar with the data and imported in ATLAS. Ti, version 7, a qualitative data management software [24]. We (NK and ESO) used six samples of transcripts to develop codes in accordance with points of care visited, reasons for the choice of point of care, outcomes from each point of care and the challenges encountered at the different points of care. The two authors then used developed codes to code and reorganize the whole data into categories by comparing statements from the respondents. Repeated/common patterns and systematic relationships guided the formation of categories and themes according to the objectives of the study.

## Results

Most of the respondents in the study were females (19) with 11 males, and were the primary caregivers closely related to the patient (Table 1). The mean age for the caregivers was 46 years with youngest at 22 and the oldest at 70. Details of the demographic characteristics of both caregivers and care receivers are indicated in Table 1 and Table 2 respectively (additional file). The study findings indicated that there was variability from individual to individual in the pathways to care undertaken by caregivers and or families of patients with Alzheimer’s disease and related dementias. The points of care varied from one to four and included; hospitals, clinics, places of religious worship and traditional healers’ shrines while six encounters/visits of these health care points were observed.

**Table 1:** Demographic characteristics of participants (*n* = 30). **Demographic characteristics of 3 respondents are missing

Demographic characteristics	Mean ranges & Categories	Numbers of participants
Gender	Females	17
	Males	10
Age (years)	18–29	6
	30–39	3
	40–49	9
	50–59	5
	≥60	4
Relationship with patient	Daughter/son to Mother	12
	Daughter/son to Father	3
	Daughter/son-in-law	3
	Grandson/daughter	5
	Husband	3
	Wife	1
Level of education	Not at all	2
	P1-P7	9
	S1-S4	6
	S5-S6	2
	>S6	8

**Table 2:** Demographic characteristics of patients with dementia (*n* = 24). **Demographic characteristics of 6 patients are missing

Demographic characteristics	Categories and Mean ranges	Numbers of participants	Total
Sex	Males	8	24
	Females	16	
Age	60–69	4	24
	70–79	3	
	80–89	8	
	90–99	6	
	100–100+	3	

There was one broader theme captured: the points of care choice with four broader categories: (i) hospitals, (ii) clinics, (iii) places of religious worship and (iv) traditional healers shrines. There were three sub-categories: facilitators/reasons for the point of health care choice, outcomes and challenges encountered at each point of health care. Apart from one respondent, the rest had visited more than one point of care. The choice for each point of care was dependent on several factors ranging from knowledge of the individual on the disease, financial status, cultural and individual/family beliefs, the outcomes from previous points of care visited and challenges experienced in the process of seeking care. The study observed that most of the caregivers and other family members (76%) didn’t know the disease and attributed the condition to normal aging process and that led to delayed healthcare seeking process. Some however, attributed it to witchcraft (demonic attacks) which caused them follow the non-formal pathways of seeking care including traditional healers and religious prayer warriors.

**Fig. 1:**
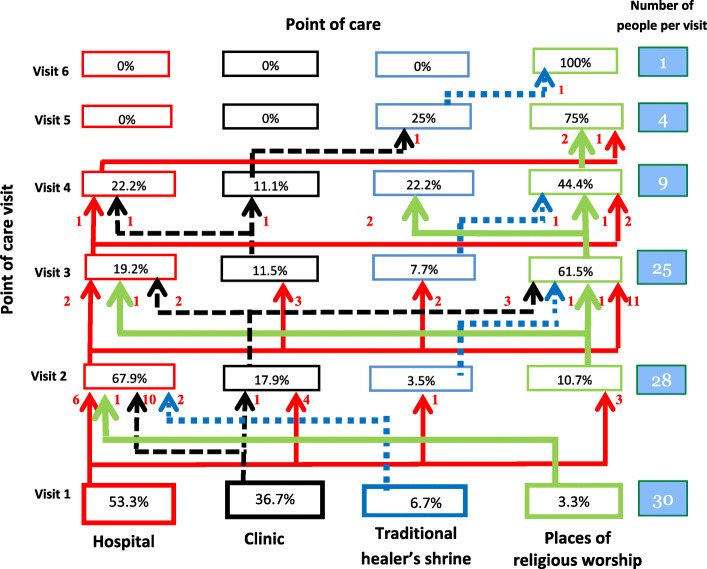
An illustration of how patients with Alzheimer’s disease and related dementias seek health care from different care providers indicating where they start from (visit 1) and where they end. Legend: Colors correspond to the point of care where patients started seeking health care. The red arrows indicate the pathway from hospital point of care. The black dotted arrows indicate the pathway from clinics; the blue dotted arrows indicate the pathway from the traditional healers’ shrines and the green arrows indicate the pathway from places of worship. Visit refers to health seeking care encounter. Numbers on the arrows indicate number of patients from one point of care to the next. Percentages in boxes indicate respondents who visited that particular point of care on that visit. Note that total number of patients/caregivers from one visit to another go on reducing as some used few health care encounters than others

Most of the respondents reported use of hospitals at first and second visit of seeking health care, but religious places of worship became more common with subsequent points of care visits while traditional healers received minimal as shown in Fig. 1.

### Hospital point of care

Of the four points of care visited, the most frequently visited point of care at initial visit was hospitals (53.3%) followed by clinics (36.7%), traditional healers (6.7%) and places of religious worship (3.3%). For hospital use, most of the respondents reported use of the facility at 1st and 2nd health seeking encounter (53.3 and 67.9% respectively) but reduced to 19.2 and 22.2% for 3rd and 4th point of care visit but none at 5th and 6th visit (Fig. 1). Facilitators, outcomes and challenges encountered at this point of health care were noted.

#### Facilitators

Several factors were said to have influenced selection of this points of care including Knowledge of the individual on the disease, stress and loss of dear ones and outcomes from previous points of care visits.

##### Knowledge of the individual on the disease

Majority of respondents who went to hospital as their first point of care did so because they thought their patients had some other illnesses and not dementia. They thought that forgetfulness was due to normal aging process which cannot be treated and so was not necessary to go to the hospital. They used terms like “ahugire”, “ajwangukire” to describe the patients, and these terms are usually used to describe the behavior of the older adults with forgetfulness. Verbatim quote;

*“That one (forgetfulness) cannot be treated, its old age, when the knowledge reduces can you rewind it (meaning there is no treatment for memory loss). We just took him to hospital because of ulcers and pressure”. (Female caregiver - wife (ID#06)).*

##### Stressing factors

Some of the respondents thought the condition was a result of stress and many thoughts from loss of their dear ones like children while others saw the condition new to them and strange. This prompted them to seek health care from trained personnel as shown by the quote”.

“*… … she got an attack which I could not manage because I had never seen that condition, I didn’t understand it. So I took a video and showed it to him (doctor) that is when he told me that it was dementia. When I reached home, I googled and found that those attacks are called seizures that are symptoms of dementia”. (Female caregiver – daughter (ID#05)).*

##### Outcomes from previous points of care visits

For those who visited hospital at their 2nd and 3rd health care encounter, the major reasons were lack of improvement (inability to manage the condition) from previous points of care visits and worsening of the condition despite all the efforts done. These finding are demonstrated by the following quotes:

*“The doctor in the nearby clinic couldn’t manage the condition and the condition was worsening, and the doctor there told us to go to the National Mental health referral hospital. We went there and admitted her for a month as I said before”. (Female caregiver – daughter (ID#024)).*

*“When we saw the condition worsening and he started to get lost, we decided to take him there (meaning hospital) so that they give him medicine and he may stay in one place but he did not respond and he continued moving aimlessly”. (Female caregiver – wife (CG#02)).*

*“We used the medicine for like two months and it did not help her, the arms even started shaking, when she failed to respond and we went to the doctor he told us that we stop the drug for two months but for me I saw that she was still in pain. When he discharged us we went to the psychiatry department”. (Male caregiver – son (CG#04)).*

#### Outcomes

In term of the outcomes of the care seeking, most of the respondents (58.1%) whose first point of care was hospital acknowledged some improvements like stopping night movements, recognizing some people, and reduced health deterioration upon visitation and condition management. However, there were few respondents who reported no improvement while others mentioned negative outcomes such as increased body weakness in patients, which they attributed to the medications. Some of the verbatim quotes are illustrated below:

*“When he started on the treatment something that could make him move at night stopped, the medicine made him so weak and became calm. … … from there he stabilized”. (Female caregiver – daughter-in-law (CG#14)).*

*“She is not completely stable but she is better than how she used to be, she can recognize a few people now”. (Female caregiver – granddaughter (CG#01)).*

The outcomes of hospital visits at the 2nd and 3rd point of care encounter seemed to be indifferent from those of initial encounter. The respondents reported some relief and improvements on the symptoms of dementia.

#### Challenges encountered

In terms of challenges encountered at hospital point of care, respondents mentioned lack of drugs as the main challenge and so had to sell their properties to meet the costs of buying drugs or and some had to take the patients back home. The respondents indicated that there was always stock out for the prescribed drugs and they were forced to buy them despite of their financial challenges as shown by the quote below;

*“At specialized hospital they asked money for buying medicine because they were not available in the hospital, to test blood and even for the CT scan but we did not get it, and he worsened and so we took him back home. We had bought medicine for the whole week”. (Female caregiver – wife (CG#02)).*

Additionally, some respondents indicated that drugs from hospitals worsened the symptoms of the condition and so ended up leaving them opting for other alternative healthcare providers. Other respondents reported rudeness as one of characteristics of health workers in the health facilities.

### Clinic point of care

#### Facilitators

The choice for this point of care was facilitated by ***proximity*** and ***physical symptoms*** of other suspected diseases. Respondents who reported to have used clinic as one of their points of care did so mainly due to proximity of these health facilities and their homes. Most of the respondents however, claimed it was primarily the physical symptoms of illness such as suspected malaria or other unspecified symptoms that compelled them seek medical care at these facilities.

*“We were not sure of the problem but we thought maybe it is cerebral malaria that’s when we took her to the clinic (name of clinic withheld) and they said they cannot manage the condition … …*” *. (Female caregiver – daughter (CG#24)).*

#### Outcomes

On to the outcomes, clinics in general seemed to offer limited or no improvement to the symptoms of the disease. The caregivers and or family heads had to continue searching for appropriate health care as indicated by the quote below;

*“Ever since he got all that medication from there (meaning clinic) he has never got any help” (improvement). (Female caregiver – wife (CG#06)).*

#### Challenges encountered

In terms of challenges encountered at clinic point of care, responses from the caregivers showed that health workers at the facility couldn’t manage the condition, and some did not even understand the mental problem. That led to poor diagnosis and management. Verbatim quote:

*“The doctor couldn’t get and understand what was exactly disturbing her and that led to treating it as mild malaria”. (Female caregiver – daughter (CG#24)).*

The challenges encountered at subsequent visits to this point of care were no different from those of visiting it at first encounter, with respondents reporting poor management or poor diagnosis of the condition.

### Places of religious worship point of care

As patients visited different points of care for help, they did so to places of religious worship at different times of health care encounters ranging from first-to-sixth unlike other types of care which received less encounters. Also the number of people who visited this point of care was twice as much as that of the hospital at third health care encounter. Whereas this point of care received minimal use at 1st and 2nd points of health care visit, it was more preferred in subsequent visits. That is; from 1st to the 6th point of health care visit in the order of 3. 3, 10.7, 61.5, 44.4, 75 and 100%. Facilitators, outcomes and challenges encountered at this point of health care are given.

#### Facilitators

There were varied reasons for use of places of religious worship in preference to the formal health care settings.

##### Cultural beliefs

The most frequent compelling factor was linkage of the condition to demonic/satanic attacks. Most of the symptoms like talking to the dead, hallucinations where relating to those known for demonic attacks. They thought through prayers they could know or chase the demons disturbing the patient.

##### Religious healing

Some caregivers and family members believed in spiritual healing (of any problem) and others thought the patients would speak out demonic voices of dead people who could be attacking them when prayed for. These finding are demonstrated in the quotes below;

*“When she tells you that in her dreams she saw dead people calling her, and like the following day she falls sick … we could call the prayer people to come and pray for her, then after the prayers she gets better. …*. *that’s when we got to know that the problem was demonic attacks” (Female caregiver – daughter (CG#26)).*

*“You know when you pray to God he listens and answers your prayers so she may get healed because of the prayers. We have hopes” (Female caregiver – daughter (CG#26)).*

*“We would pray that a miracle happens and see her getting healed” (Female caregiver – daughter-in-law (CG#03)).*

*“Why we went for prayers, they (meaning other people) told us that when a person has such mental illness and is prayed for she may fall down and speak if they are demons and you can be able to know what is bothering her” (Female caregiver – daughter (CG#11)).*

##### Poor outcomes from previous points of care

Some of the caregivers who used places of religious worship at 2rd point of health care encounter and onwards, reported lack of improvement from health facilities they had visited before, and seemed to have lost hope on patient recovery from those facilities. For that reason caregivers had to resort or take refuge to religious prayers as indicated by the verbatim quote;

“*The patient was not getting cured; the church people would come here and pray for us, tell us to be patient with her and keep taking care of her until God calls her” (Female caregiver – daughter-in-law (CG#07)).*

#### Outcomes

When asked on the outcomes from places of religious worship, respondents reported to have seen no any improvement in the symptoms of the disease, and thought the symptoms are just part of normal aging process. Verbatim quote:

“*…*. *that one (meaning forgetfulness) is old age that has no medicine and there is no cure for it”. (Female caregiver – daughter (CG#26)).*

Despite lack of improvement reported at this point of health care, positive complements and hence emotional support have been attached to places of religious worship as one respondent reported below;

*“There was no improvement, they would come to comfort us to be patient, and on how we can take care of her and not regretting”. (Female caregiver – daughter-in-law (CG#07)).*

On the other hand some caregivers reported use of both places of religious worship and hospital in tandem, and so were not sure of what caused observed improvement in the symptoms of the condition.

#### Challenges encountered

When asked on the challenges they encountered at this point of health care, the caregivers generally reported no challenges experienced during the health care seeking process with places of religious warship, save lack of improvement in the symptoms of the disease.

### Traditional healer’s shrine point of care

This point of health care was visited at different levels of health care encounter ranging from1^st^ to 5th but not in 6th point of care encounter.

#### Facilitators

The choice for this point of health care was facilitated by lack of knowledge on the disease, cultural beliefs in demonic attacks and spiritual healing, and poor outcomes from previous points of care.

##### Lack of knowledge of the disease, cultural/individual belief in demonic attacks and spiritual healing

Most of the respondents whose first point of care was traditional healers’ shrine reported to have never seen such kind of condition and so took their patients to the healers because they believed their patients had family issues (meaning demonic attacks) and would be healed by the traditional healers. Those who visited this point of care in subsequent point of care encounters mentioned similar compelling factor of being confused with the disease and thought it was due to family problems such as witchcraft. The quotes below demonstrate the findings.

“*… .we first went to traditional healers because we had not known the disease; this disease in its initial stages its confusing, for us we thought that they were traditional things* (meaning witchcraft*) …*” *. (Female caregiver – wife (CG#10)).*

*“Staying with someone and he or she starts doing uncoordinated/wrong things, people in the village convinced us that it is about family problems which need traditional healers”. (Female caregiver – daughter (CG#11)).*

##### Poor outcomes from previous points of care

Caregivers who did not meet their expectations of cure from initial points of care like formal health facilities looked for alternative means of helping their patients. They were particularly going to witchcraft for spiritual healing or herbalists for herbal medicine.

“*… ..because he (meaning patient) usually hears those adverts from the radios he would go and buy the herbal medicine for himself”. (Female caregiver – wife (CG#19)).*

#### Outcomes

In terms of outcomes, all respondents who visited the traditional healer’s shrines were very disillusioned with the results. They went there with high expectations but none of them reported any help. Majority believed that traditional healers were liars and had no skills to manage the symptoms of dementia. Verbatim quote;

*“For sure I will be deceiving if I say they helped us. We didn’t get any help from witchdoctors instead he (meaning patient) almost lost his life from there”. (Female caregiver – wife (CG#10)).*

##### Challenges encountered

When the respondents were asked the challenges they encountered in the process of seeking care at the traditional healers’ shrine, their responses indicated that it was wastage of time and money as their medicine did not help their patients at all. The response from one of the caregivers among those who visited this point of care was not impressing at all as shown by the quote:

“… … *he wanted us to sit on skins of certain animals of which I never accepted … then we sat on normal sits in the sitting room and he told me that since I have rejected to do his things my grandmother will not get healed.... he wanted us to be cut and put his herbs in our blood but we refused, it was not easy”. (Female caregiver – granddaughter (CG#28)).*

## Discussion

The study explored pathways to hospital care undertaken for patients with Alzheimer’s disease and related dementias and also challenges experienced in the process of seeking care. The pathways to care varied considerably from individual to individual due to the interplay of various factors. Six visits/encounters of health care points were observed and the four points of care visited were; hospital, clinic, traditional healer’s shrine and places of religious worship. Each caregiver would visit at least one to four of the health care points once or more with varied reasons for each point of care. Most of them indicated use of hospital point of care at initial and second point of health care visit and numbers reduced gradually. While places of religious worship, clinics and traditional healers received few patients at initial visit, places of religious worship became more common with subsequent care visits. Reasons and outcomes together with challenges encountered for each point of health care are discussed. Knowledge gap on the disease, peoples’ beliefs in spiritual healing, lack of expected outcomes at different points and other challenges encountered like financial incapacitation and management incapability influenced the health care pathways. Major improvements were observed on hospital use but little or no help with other points of care visited.

While hospital dominated as the first and second point of health care, it was minimally used at fourth and completely unvisited in the fifth and sixth health care encounters. High use of hospitals at initial (and second) points of care visits indicate strong belief people have in the formal sector particularly at hospital level. Indeed people expect health professionals to handle various ailments at a hospital Handley, Bunn [25], and this expectation tallies with the observed improvements (58%) in the symptoms of dementia at the hospital level.

The opting out of hospital use for non-formal care setting like places of religious worship was a consequence of several factors including knowledge gap on the disease. The study found that 76% of the caregivers attributed the disease to normal aging process, a scenario that has been reported in various studies including a parallel study done in the same area of Southwestern Uganda on caregivers and patients perceptions about Alzheimer’s and related dementias [26]. Other studies have also documented this knowledge gap on the disease in other countries including the neibouring Tanzania [21] and the USA [27]. Such knowledge gap caused the caregivers not seek medical care for their patients early in time or did so while treating “other” conditions or kept meandering in the non-formal care pathways.

Also the rare manner in which symptoms of dementia presented to the caregivers for example bad dreams, hallucinations, irrational talks, etc., resembled demonic attacks [28], and so caused them opt for spiritual prayers or traditional healers. Such people’s belief in spiritual healing has been reported in several countries both developed and developing world [26, 29, 30]. Similarly, the explosion of Pentecostal churches and other prayer warriors in sub-Saharan Africa or in populations of African descent has made religious healing one of the most popular interventions for psychiatric and neurologic disorders [28] and emotional support [31]. It has also been shown that religion and hence prayers becomes increasingly important in times of life-threatening illness not only in Uganda [26] but also in developed world [30].

While 58% of the respondents reported improvement in the symptoms of the disease, others reported lack of improvement in the quality of life for dementia patients at the hospital as they expected, and so had to shift their pathway of seeking health care from formal care settings to non-formal as earlier reported by Salgado, Lord [32].

The barriers/challenges encountered at the hospital point of care influenced the caregivers to opt for alternative non-formal pathways like places of religious worship and traditional healers’ shrine. The major challenge experienced by most participants at hospital level was lack of drugs at the facilities due to stock-out for prescribed drugs. This is common in Ugandan health systems particularly in government facilities [20]. This had a financial implication on the side of caregivers. Whereas some could dig deeper into their pockets or sell property to meet financial needs of buying prescribed drugs outside hospitals, maintenance during hospitalization and other expenditure involved, others could not manage and had to resort to other means of care support such as religious prayers. That explains the increased number of religious prayer visits after two formal health care encounters.

The reported challenge of worsening effects of treatment of the disease was not observed for the first time. In a study by Chidgey [33] many family caregivers were convinced that hospital care caused deterioration in people with dementia. Such negative outcomes of treatment from the hospital has been attributed to medication errors by the patients or caregivers themselves especially with the older adult population [34], by contraindication of the drugs [35], but majorly a health professionals’ responsibility [36]. In their study, Pham and colleagues (2011) found that physicians in the USA were responsible for 24% of errors and nurses at 54%, with most of these errors commonly occurring in the administration phase (36%) through improper dosage/quantity (18%), not following right procedure/protocol (17%) or because of poor communication (11%) [36]. This agrees with Harwood [37] who asserts that if treated in the wrong way, people with dementia are easily distressed, suffer complications and may be unnecessarily disabled. Also poor response after multiple treatment options in patients with dementia has been reported [38] even with adverse central effects especially when using opioid analgesic drugs [35]. At clinic level, the observed minimal visits to clinics are well understood and expected. While proximity to these facilities led some people to visit them for treatment of other ailments, lack of trained health care professionals to handle mental health disorders at clinics/health centers is another barrier behind low visitation of this care point. At a global perspective, most of the clinics in rural setting are managed by nurses and clinical officers who are not well trained to handle complicated mental disorders like dementia that need specialized medical attention [39]. This is consistent with Uganda’s Ministry of Health strategic plan [8] where specialized clinical services for mental health disorders are only provided at regional referral hospitals. Moreover, these regional referral hospitals receive patients from the lower health facilities such as clinics and health centers.

In the non-formal pathways to health care, use of traditional healers shrines was more witnessed in rural areas than semi-urban or urban areas, agreeing with Tumuhairwe, Maling [26] study in the area. This scenario that has previously been reported in Nigeria, and is a consequence of knowledge gap and individual/ cultural beliefs [40].

At places of religious worship, the minimal challenges experienced are linked to devotion and religious belief people have in spiritual healing [29], and so influenced the formal to informal care shift in search of health care. This is in line with the statement from contemporary Nigeria by Agbaje and Babatunde [41] who stated that “every ailment has spiritual implications and that drugs alone are not adequate,” meaning that medicine alone is not enough for treatment of dementia and other neurological and psychiatric symptoms Agbaje and Babatunde [41].

Differences however, exist in caregiving and hence quality of life for people with dementia between developed and developing world. For example, in developed countries, there are some structured yet individualized approaches used to train family caregivers to reduce behavioral and psychiatric disturbances in people with Alzheimer’s disease by teaching caregivers to monitor problems, identify possible events that trigger disturbances, and develop more effective responses [42]. This is lacking in health systems of developing countries like Uganda yet the approach has proved to be successful in improving caregivers’ quality of life which is always under looked in the process of caregiving, and also in reducing dementia-related problems including depression [43], agitation and sleep disturbance [44].

Also use of technology-based interventions for caregivers like use of computers, telephones, e-mail, and the Internet to provide support and information to informal caregivers has been demonstrated to be effective in developed countries [45] but are yet to be adopted in the developing world. However, one respondent (Female caregiver – daughter (ID#05)) demonstrated use of telephone to record a video clip showing patient behavior which she took to the doctor who diagnosed dementia from the clip. This confirms the effectiveness of technology in caregiving process, but is still underutilized in developing countries.

Overall, the observed healthcare challenges/barriers including limited knowledge of the disease, cost of prescribed drugs, perception of lack of efficacy of the drugs, lack of compassion from health workers, limited expertise in specialized care for mental health, low availability of health facilities with mental health specialists, unexpected outcomes from the health facilities well fits the [18] healthcare access barriers mode, which categorizes these factors as financial, structural and cognitive. These barriers have a greater impact on under-utilization of formal health care services for patients with dementia [46] through decreased screening, late presentation to healthcare and lack of treatment, which in turn result in poor health outcomes and health disparities among the patients with dementia [18]. It is therefore clear that, studying the pathways to care is critical to describing health services utilization, characterizing of the sources of delay in attending the right care, and can be used to identify the possible remedies for improved healthcare outcome in patients with dementia.

### Limitations

The study only covered those who had visited hospitals or received formal diagnosis leaving out those meandering in the informal care settings and have not reached hospital point of care. The study also did not specify the time period over which the multiple points of care were identified.

## Conclusions

Study findings indicate that caregivers and or families of patients with dementia go to both formal and informal care settings to seek health care for their patients. Although findings indicate lack of a specific pathway to care for the patients with Alzheimer’s disease and related dementias, most of them begin with hospital (formal) and end with non-formal points of care. Most caregivers visit hospitals in their first health care visits not for treatment of dementia as per say but other ailments while places of religious worship gain popularity in subsequent points of care visits. This pathway is facilitated by several factors including limited knowledge on the disease, financial incapacitation, cultural beliefs, family/individual belief in spiritual healing and poor outcomes from the formal health care settings. The limited use of hospital point of care after initial visits was a consequence of unavailable medications and its deterrent effect of selling family properties to meet the costs of buying drugs, and so have caused some patients seek care from the informal health care providers.

### Recommendation

Health systems need to design and promote programs aimed at raising awareness about Alzheimer’s diseases and related dementia. People need to know that dementia is a manageable disease and not part of normal aging process.

## Supplementary information

**Additional file 1.** Interview guide used for data collection.

**Additional file 2.** Code list generated after analysis with ATLAS.Ti version 7.

## Data Availability

The datasets generated and/or analysed during the current study are available in the supplementary information files.

## Abbreviations

AD/RDs: Alzheimer’s disease and Related Dementias
MADRI: Mbarara Alzheimer’s Disease and Related Dementias Initiatives
MUST-REC: Mbarara University of Science and Technology Research Ethics Committee
NIH: National Institutes of Health
UNCST: Uganda National Council for Science and Technology
LMICs: Low- and middle-income countries
