# Supplementary material for: Exploring pathways to Hospital Care for Patients with Alzheimer’s disease and related dementias in rural South Western Uganda
Source: BMC Health Serv Res. 2020 Jun 3;20:498. doi: 10.1186/s12913-020-05365-5 (PMC7268702; doi:10.1186/s12913-020-05365-5)
Supplement: Supplementary file 1 — Additional file 1. Interview guide used for data collection. [file 12913_2020_5365_MOESM1_ESM.docx]

**Additional file 1: Interview guide**

**Code: _______________________________**

I would like to appreciate your willingness to participate in this study and for giving us your time.

I am NATHAN KAKONGI undertaking a study on pathways to hospital care for a disease of forgetfulness related to ageing in South-western Uganda.

We need you to share with us about what people with disease of forgetfulness go through while seeking health care. Disease of forgetfulness (Dementia) is a disease that comes with forgetfulness in old age. The information you give us will be kept confidential and will only be used for purposes of this study. This interaction will take not more than 1 hour. In order not to miss any information we shall also record your answers. Kindly be audible enough so that we don’t miss any of your answers. OK?

**Personal background**

I understand you are caring for a person with this problem of forgetfulness.

• How are you related to the patient?

• Do you stay with him/her?

• How long have you been staying together?

• What about caring for him/her?

• How old are you?

**Health care seeking behaviour**

1. When you realized that your patient had a problem what did you do?

2. Where did you first go for help? (probe if it was health facility, traditional healers, religious institution, etc)

3. How long did it take to get the diagnosis of disease of forgetfulness? (probe for where the diagnosis was received from, and source of referral)

4. Other than medical, where else had you go for help? (probe until you have exhausted all the sources of care used including traditional healers, religious institutions)

5. Where did you go first, second, third? (Probe until you have exhausted the steps taken)

6. If they have used more than one source of care, ask for reasons)

**Interactions with formal and informal care systems**

1. When you went to the facility, whom did you see? (Start with where they went first.)

2. What did they do to help the patient?

3. What kind of information did you receive concerning the disease?

4. Do you get what you expected from there?

5. Did you get satisfied with what was done?

6. How was the patient’s condition after the visit?

**Challenges / problems met while seeking care**

1. Did you meet any problems/challenges/disturbances while seeking care in all those places?

2. What kind of challenges did you meet in those places? (probe until the list of challenges is exhausted)

3. How did you deal with these challenges?
