# Supplementary material for: Exploring pathways to Hospital Care for Patients with Alzheimer’s disease and related dementias in rural South Western Uganda
Source: BMC Health Serv Res. 2020 Jun 3;20:498. doi: 10.1186/s12913-020-05365-5 (PMC7268702; doi:10.1186/s12913-020-05365-5)
Supplement: Supplementary file 2 — Additional file 2. Code list generated after analysis with ATLAS.Ti version 7. [file 12913_2020_5365_MOESM2_ESM.rtf]

Code-Filter: All
______________________________________________________________________

HU:	ATLAS.Ti Analysis
File:	 [D:\MADRI\ATLAS.Ti Analysis.hpr7]
Edited by:	Super
Date/Time:	2020-03-27 10:41:09
______________________________________________________________________
Social-demog
Reasons for pc5
reasons for PC4
Reasons for pc1
Reason for pc6
Reason for pc3
Reason for pc2
Point of care2
Point of care 6
Point of care 5
Point of care 3
Point of care 1
Point care 4
perceived cause
Outcome at pc5
Outcome at pc4
Outcome at pc3
Outcome at pc2
Outcome at pc 1
Influencing factors
Response to the med..
Factors
Disease identity
diagnosis uncertainty
Challenges in pc1
Challenges in pc 5
Challenges in pc 4
Challenges in pc 3
Challenges in pc 2
Challenges
